# The fungal mycobiome: a new hallmark of cancer revealed by pan-cancer analyses

**DOI:** 10.1038/s41392-023-01334-6

**Published:** 2023-02-01

**Authors:** Zhi Zong, Fangfang Zhou, Long Zhang

**Affiliations:** 1 International Biomed-X Research Center, Second Affiliated Hospital of Zhejiang University School of Medicine, Zhejiang University, 310058 Hangzhou, China; 2 Institute of Biology and Medical Science, Soochow University, 215123 Suzhou, China; 3 MOE Key Laboratory of Biosystems Homeostasis & Protection and Innovation Center for Cell Signaling Network, Life Sciences Institute, Zhejiang University, 310058 Hangzhou, China; 4 Cancer Center, Zhejiang University, 310058 Hangzhou, China

**Keywords:** Genome informatics, Tumour biomarkers

In a recent study published in *Cell*,^1^ a group led by Ravid Straussman and Rob Knight characterized fungi across multiple types of cancer and revealed their distribution, relationships with immune cells, and possible prognostic value. Meanwhile, another study led by Anders B. Dohlman and Iliyan D. Iliev^2^ reported a similar pan-cancer mycobiome analysis of diverse body sites and identified tumor-associated fungi.

For more than a century, researchers have been exploring the links between cancer and microbes such as bacteria and viruses;^3^ however, only few work have focused on the relationship between fungi and cancer. Much of the research performed in recent years has focused on the human microbiome, especially the gut, where bacteria, viruses, and fungi are more abundant and diverse than anywhere else in the body. Although fungi have been reported to promote tumorigenesis,^4,5^ the role and effects of cancer-related fungi are still largely unknown.

To initially characterize the cancer mycobiome, Narunsky-Haziza et al.^1^ analyzed the internal transcribed spacer (ITS) sequencing and whole genome sequencing (WGS) data of tissue, blood, and plasma-derived from over 17,000 patients across 35 cancer types from four international cohorts. By using sequencing data from Weizmann (WIS) and The Cancer Genome Atlas (TCGA), the authors profiled the fungal DNA and defined the fungal signature associated with diverse cancers. Further, they integrated four staining techniques with varying principles to visualize as many fungi as possible in different cancer tissue microarrays. The images showed that fungi are ubiquitous across cancers and the compositions of fungi are cancer type-specific. Similar to the gut microbiome, the abundance of fungi was lesser than that of bacteria in tumor tissues. Mycobiome richness showed significant differences across cancer types in these cohorts, and the fungal expression was markedly higher in tumor samples than in controls. For example, *Cladosporium sphaerospermum* and the *Cladosporium* genus were found enriched in breast tumors; *Aspergillus* and Agaricomycetes were elevated in lung cancer. Notably, the authors developed a strategy that can distinguish patients with cancer and healthy individuals by defining a signature of circulating fungal DNA from 20 different kinds of fungi. This suggests that the mycobiome may have diagnostic value in cancers even in the early phase of the disease.

**Fig. 1:**
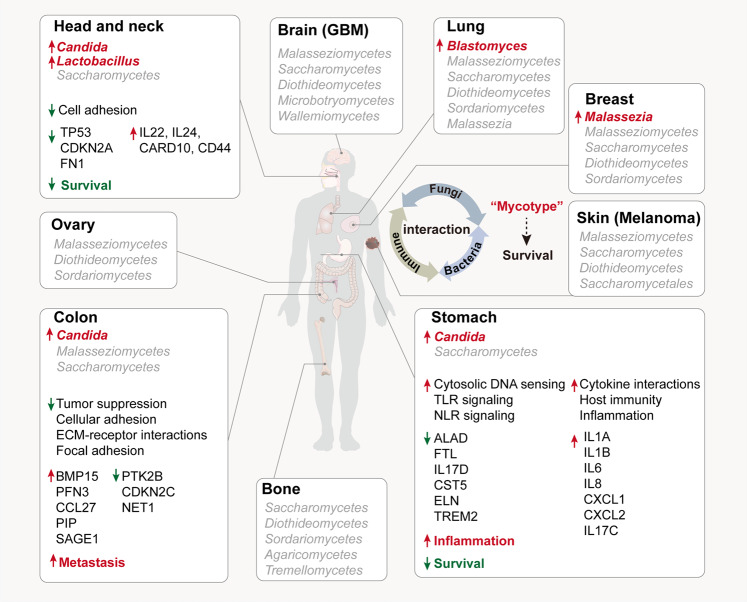
Cancer-associated fungi found in different body sites and gene expression signatures. Fungi found to promote tumorigenesis in different body sites (lung, breast, skin, brain, bone, ovary, colon, head-and-neck, stomach) are listed. Fungi that were highly enriched in cancers and may serve as potential prognostic markers are highlighted in red. The presence of *Candida* in head-and-neck and colon tumors appears to be associated with pro-tumorigenic and cellular adhesion-related gene pathways, whereas in stomach tumors it seems to be involved in inflammatory responses that ultimately lead to poor prognosis. Multiple fungal-bacterial-immune ecologies were detected across tumors. These mycotypes may be promising to predict the survival of patients with cancer

Given the physical interactions between fungi and bacteria, the authors sought to investigate the interaction between the mycobiome and bacteriome in the context of the tumor. They found that most types of fungi have specific bacterial species with which they tend to co-exist. This means that tumors may favor the growth of both fungi and bacteria, thereby forming communities with non-competitive ecologies—unlike typical environments where fungi and bacteria compete for shared resources. Using multiple modified machine learning strategies and differential abundance testing of mycobiomes, the authors could discriminate between cancer and non-cancerous tissues and within cancer types, which underlies the diagnostic and prognostic potentials of the cancer mycobiome (Fig. 1).

In another story,^2^ the authors performed an analysis using WGS data from various cancer samples. In line with Narunsky-Haziza et al., Dohlman and colleagues also revealed that, compared to bacterial DNA, fungal reads accounted for a much smaller proportion of microbial reads. In addition, tissues from the head and neck, colon and rectum, and stomach showed a relatively higher abundance of fungal DNA when compared to those from the esophagus and brain. Fungal DNA can originate from not only tumor samples but also from contamination. Therefore, it is crucial to remove contaminant fungi that may lead to false-positive signals. To this end, the authors utilized a prevalence-based decontamination model that enables the precise capture of the mycobiome from the samples.

When it came to the compositions of fungi in cancers, they found that gastrointestinal (GI) cancers differed in their richness of *Candida* and *Saccharomyces* and can thus be classified into *Candida*-dominant (*Ca*-type) and *Saccharomyces*-dominant (*Sa*-type) tumors. Fungal infection is often associated with T helper 2 (T_H_2)-type allergic reactions. Interestingly, these two types of GI cancers showed different gene expression patterns and immune responses. For instance, *Ca*-type tumors were linked with the overexpression of pro-inflammatory cytokines such as interleukin (IL)-1 and IL-6, suggesting a Type 17 signature. Unlike *Candida*, which was observed mostly in stomach cancers, *Blastomyces* were dominant in lung tumor samples, and *Malassezia* was prevalent in breast cancers. In addition, the presence of *Candida* was regarded as a predictive biomarker of late-stage disease in GI cancers, and was associated with poor survival (Fig. 1).

Overall, these two pan-cancer studies suggest the potential impact of fungi on host immune response and cancer progression, supporting the notion that the microbiome, including the mycobiome, is a key part of cancer biology. The findings also unveil the co-existence and close associations between fungi and bacteria. This should spur further attention to study their synergistic roles in tumors. Of note, these new findings provide potential translational implications, not only in cancer diagnosis and prognosis but also in other aspects such as drug development.

Limitations of these studies lie primarily in the sample pools, as majority of the samples the authors collected were not specifically for microbiome studies. Thus, researchers need to carefully eliminate possible contaminants and false-positive fungal DNA, and repeatable results are needed using samples obtained from a germ-free environment. While these studies provide an explicit link between cancer and fungi, more research is needed to understand the underlying mechanisms by which fungi induce inflammation and promote cancer progression. To answer these questions, researchers need to study one type of cancer at a time and use cells and animal models to examine whether fungi drive healthy cells to become cancerous. Once researchers gain a more comprehensive picture of how fungi function in cancer, they may be able to develop antimicrobial therapies or targeted pre- or probiotic therapies that benefit patients with cancer.
